# Galectin-3 Mediates Endothelial-to-Mesenchymal Transition in Pulmonary Arterial Hypertension

**DOI:** 10.14336/AD.2018.1001

**Published:** 2019-08-01

**Authors:** Tangzhiming Li, Lihuang Zha, Hui Luo, Suqi Li, Lin Zhao, Jingni He, Xiaohui Li, Qiangqiang Qi, Yuwei Liu, Zaixin Yu

**Affiliations:** ^1^Department of Cardiology, Xiangya Hospital, Central South University, Changsha, China; ^2^State Key Laboratory of Cardiovascular Disease, Fu Wai Hospital, National Center for Cardiovascular Diseases, Chinese Academy of Medical Sciences and Peking Union Medical College, Beijing, China; ^3^Centre for Pharmacology and Therapeutics, Division of Experimental Medicine, Imperial College London, Hammersmith Hospital, London W12 0NN, UK; ^4^Department of Pharmacology, School of Pharmaceutical Sciences, Central South University, Changsha, China; ^5^Department of Cardiology, Shenzhen People's Hospital, the First Affiliated Hospital of Southern University of Science and Technology, the Second Clinical Medical College of Jinan University, Guangdong, China.

**Keywords:** Galectin 3, endothelial-to-mesenchymal transition, pulmonary arterial hypertension

## Abstract

Galectin-3 (Gal-3) is highly expressed in fibrotic tissue related to diverse etiologies. endothelial-to-mesenchymal transition (EndoMT), A less well studied phenomenon serves as a critical process in pulmonary vascular remodeling associated with the development of pulmonary arterial hypertension (PAH). EndoMT is hypothesized to contribute to the over-proliferation of αSMA positive cells. We aim to investigate the potential role of Gal-3 in regulating EndoMT in PAH. We observed an upregulation in both Gal-3 and αSMA expression in the monocrotaline (MCT) and Hypoxia PAH model, accompanied with intimal thickening. For more profound vascular remodeling and endothelial layer lesion in former model, we employed Gal-3 knockdown and overexpression lentivirus methodology to the MCT rats to determine the mechanisms underlying abnormal endothelial cell transition in PAH. PAH was evaluated according to right ventricular systolic pressure, right heart hypertrophy and pulmonary artery remodeling. A reduction in Gal-3 was protective against the development of PAH, while Gal-3 upregulation aggravated pulmonary vascular occlusion. In addition, Gal-3 deficiency suppressed pulmonary vascular cell proliferation and macrophage infiltration. Finally, we revealed that in endothelial cells treated with tumor necrosis factor α and hypoxia (representing an *in vitro* model of PAH), inhibition of Gal-3 by siRNA was able to abolish the associated upregulation of αSMA. These observations suggesting Gal-3 serves as a critical mediator in PAH by regulating EndoMT. Inhibition of Gal-3 may represent a novel therapeutic target for PAH treatment.

Pulmonary arterial hypertension (PAH) is a chronic and progressive disease leading to increased pulmonary vascular resistance, right ventricular failure and is eventually fatal [1]. The pathologic changes associated with PAH are characterized by obliteration of distal pulmonary arteries, extensive muscularization of the arterial wall and precapillary pulmonary arterial loss leading to pulmonary artery (PA) remodeling [2].

Remodeling of pulmonary vessels is determined by vascular cells phenotype changes including myo-fibroblasts, smooth muscle cells and endothelial cells excessive proliferation and apoptosis resistance, especially associated with an increased number of α-smooth muscle actin (αSMA) expresses cells [3], which lead to medial thickening and obliteration of arterioles. According to the consensus view, smooth muscle cells over reproduction is the main driver of the increased production of αSMA-positive cells found in vascular lesions. However, there is an increasing evidence to suggest that endothelial cells [4-7] have the ability to transition into mesenchymal cells and co-express endothelial markers and αSMA. *In vitro*, endothelial cells exposed to chronic hypoxic stressors and inflammatory factors can undergo endothelial to mesenchymal transition (EndoMT), a process which may contribute to the accumulation of smooth muscle-like cells in vascular pathologies.

EndoMT is a biological process in which endothelial cells change their endothelial phenotype into a mesenchymal or myofibroblast phenotype. The increased migratory ability of transitional endothelial cells results in the loss of tightly cohesive cell-cell contact and makes endothelial cells move toward the inner layer of vessel[5, 6]. EndoMT often developed under unfavorable stimulus (inflammation and hypoxia, commonly). von Willebrand factor (vWF), VE-Cadherin and CD31/PECAM-1, are specific membrane proteins of endothelial cells related to cell-cell junctions and these are frequently co-express with mesenchymal markers like αSMA in EndoMT. EndoMT has been implicated in the pathology of PAH in humans[5, 6] where transitional endothelial cells play an important role in the advanced thickening of αSMA-positive cells.

Galectin-3 (Gal-3) is a β-galactoside binding lectin that belongs to the galectin family [8] and is characterized by a carbohydrate recognition domain that is highly expressed in fibrotic tissues. Gal-3 mediates the inflammatory and fibrotic responses involved in liver[9] and kidney [10, 11] fibrosis, and upregulation of Gal-3 is a strong predictive biomarker of heart dysfunction [12, 13]. Recently, Gal-3 has been suggested as an essential mediator of TGF-β [14] induced lung fibrosis through epithelial mesenchymal transition (EMT), an invasive process involving repression of the epithelial junction protein E-cadherin and upregulation of mesenchymal genes. These studies imply that Gal-3 could regulate fibrosis through EMT.

In our previous studies [15, 16], we found Gal-3 upregulation in PAH patients, and in animal experiments found that Gal-3 mediates right ventricular fibrosis and pulmonary adventitial fibroblast activation. Therefore, we hypothesize that Gal-3 is involved in EndoMT related pulmonary vascular remodeling, resulting in accelerated reduction in peripheral pulmonary arterial diameter, and ultimately contributing to the onset and maintenance of PAH.

## MATERIAL and METHODS

### Animal experiments

The experiments were approved by Institutional Animal Care and Use Committee of Central South University. Eight-week-old, weight matched male Sprague-Dawley (SD) male rats were used for experiment data.

For the monocrotaline (MCT)[17, 18] model, animals were randomized into 4 groups (n=5): Negative Control (Neg Con) group, MCT + Neg Con group, MCT+Gal-3 knockdown (Gal3KD) group and MCT+Gal-3 overexpression (Gal3OE) group. the Gal-3 knockdown and overexpression sequence were shown in Supplementary Table 1. 50ul of lentivirus suspension containing 2 × 10^8^ TU/ml lentivirus (Negative control, Negative control, Gal3KD and Gal3OE containing sequence separately) was intratracheally delivered to each rat at a rate of 25 μl/min after anaesthesia (anaesthetized by 2% Pentobarbital 30mg/kg). Seven days after lentivirus injection into the lungs of each group, MCT (60 mg/kg) was injected into the abdominal subcutaneous tissue of MCT+ Neg Con, MCT+Gal3KD and MCT+Gal3OE groups and saline was given to Neg Con group as placebo for 21 days.

For the hypoxia rat model, SD rats were randomly divided into two groups: the normoxia group and the hypoxia group. The normoxia group was kept in a normoxia environment (21% O_2_). The hypoxia group was exposed to hypoxic conditions (10% O_2_) using a ventilated chamber (Chang Jin Technology Co., Ltd., Changsha, China) for 4 weeks as described previously [16].

### Cell culture

Human pulmonary artery endothelial cells (PAECs) were purchased from Lonza (Basel, Switzerland). PAECs were cultured in EGM-2 with 2% FBS (Lonza), according to the supplier’s instructions. Synchronized cells were treated with or without TNFα (5 ng/ml or 10 ng/ml, PeproTech, US). For experiments conducted under hypoxic conditions, synchronized cells were cultured in a hypoxic incubator (O_2_ Density 0.5%) for 48 hours.

### Transfection with siRNA

siRNA for Gal-3(targeted siRNA sequence: GCAATAC AAAGCTGGATAA) and non-targeting control siRNAs (Ribobio Co. Guangzhou, China) were introduced into subconfluent PAECs at a concentration of 50nM using ribo*FECT*™ CP Transfection Kit (RiboBio) according to manufacturer instructions. We measured mRNA (data not shown) at 48h and protein levels at 72h.

### Morphometry analysis, Immunohistochemistry and immunofluorescence

Formaldehyde was used to fix the tissue and paraffin-embedded lung tissue sections were stained with hematoxylin and eosin. Morphometric analyses were performed in pulmonary arteries with an external diameter of 50-100 μm, the medial thickness was calculated by following formula: medial thickness (%) = medial wall thickness / external diameter × 100 [19]. For quantitative analyses, 30 vessels of each rat were counted, and the average medial thickness was calculated. At × 200 magnification, 30 small pulmonary vessels of each animal less than 50μm in external diameter were evaluated for muscularization. Peripheral vessels <75 μm diameter were counted at ×200 magnification. We conducted elastic staining according to the manufacturer’s protocol (Sigma-Aldrich Elastic Stain Procedure No. HT25). 30 small pulmonary vessels of each rats ranging from 10 to 50 mm in external diameter were counted for muscularisation. For immunohistochemistry examination, lung sections were stained for anti-Ki67 and anti-CD68 to evaluate proliferation and inflammation of each group. For double immunofluorescence, the sections were incubated with the primary antibodies against anti-von Willebrand factor (vWF) / anti-αSMA, anti-Gal3/anti-αSMA, or anti-Gal3/anti-vWF. Cell Nuclei was stained by DAPI.

### Knockdown and Overexpression of Galectin-3 by Lentivirus transduction

For virus transduction, PAECs were plated at ~70-80% confluency and transduced at MOIs of 20. Cells were grown for an additional 48 h in serum-containing media and were then used for the assays indicated. To knockdown or overexpress Gal-3, PAECs were transduced with Gal-3 shRNA or Gal-3 overexpression sequence, and scramble shRNA was treated to control group. 48h after transduction, cells were serum starved for 24 h, followed by stimulation with 5% FCS. The cells were harvested for western blotting to confirm efficiency of knockdown and overexpression.

Three RNAi candidate target sequences to Gal-3 were designed by GeneChem (Shanghai China). Gal3KD (Lgals3-RNAi 56032-1) had the best interference efficiency in endothelial cells revealed by Western blot and was selected to knockdown the endogenous Gal-3 in endothelial cells. Overexpression of Galectin-3 lentivirus: Gal3OE (LV-LGALS3 7255-1) was also produced by GeneChem and Gal-3 was increased after transfection of endothelial cells compared to Negative Control (Neg Con)-siRNA, as tested by western blot. The oligonucleotides encoding the Gal3KD, Gal3OE and Neg Con sequence and a loop sequence separating the complementary domains, were synthesized and inserted into the pGCL-GFP. The recombinant virus was packaged using Lentivector Expression Systems (Shanghai GeneChem).

### Lung sample preparation

Following 21 days exposure of MCT or 28 days of hypoxia, rats were anesthetized with 2% Pentobarbital (30mg/kg) and right ventricular systolic pressure (RVSP) was measured by right heart catheterization. Rats were then sacrificed, and the hearts and lungs were harvested. Right ventricular hypertrophy was determined as the ratio of right ventricular to left ventricular and septal weight (RV/(LV+ S)). The right lung was snap frozen in liquid nitrogen. The lung tissues were flushed with saline, and the right lungs from each group were isolated and preserved in liquid nitrogen for subsequent Quantitative real-time PCR and Western blot experiments. The left lungs were fixed with 4% paraformaldehyde solution for 24 hours applied for further histology and immunofluorescence staining as described below. Investigators performing all cardiopulmonary phenotyping procedures and histological analyses were blinded to animal treatment group.

Epitope retrieval was performed by boiling the sections in citrate buffer, pH 6.0. Sections were reacted with hydrogen peroxide to block endogenous peroxidase, washed, and blocked with 5% bovine serum albumin. For immunohistochemistry examination, lung sections were stained for anti-PCNA (1:200; Cat#10205-2-AP, Proteintech, USA), anti-CD68 (1:500; Cat#GB11067, Servicebio, China), with appropriate horseradish peroxidase-conjugated secondary antibodies (1/200). The number of PCNA+ and CD68+ cells was quantitatively evaluated in each high magnification field of PAs, for 12 separate fields.

### Immunofluorescence

For double immunofluorescence, the lung sections and endothelial cell underwent indicated stimulus were then incubated with the primary antibodies against anti-Von Willebrand Factor (vWF) (1:400, Cat#ab6994, Abcam, UK), anti-αSMA (1: 200, Cat#17521-1-AP, Proteintech, USA), anti-αSMA (1: 200, Cat#ab7817, Abcam, UK), anti-Galectin3 (1:100, Cat#14979-1-AP, Proteintech, USA), anti-Galectin3 (1:200 Cat#Ab2785, Abcam, UK) overnight at 4°C. the lung sections were treated with vWF/αSMA, vWF/Gal-3 or αSMA/Gal-3 co-staining. the second antibody were applied as follows: CY3 conjugated goat anti-rabbit IgG (1:200, Cat#P0183-1, Beyotime, China), Alexa Fluor 594 labeled goat anti-mouse IgG (1:200 Cat#R37121, Thermo fisher, USA), Alexa Fluor 488 labeled goat anti-Rabbit IgG (1:200 Cat# R37116, Thermo fisher, USA), and Alexa Fluor 488 labeled goat anti-mouse IgG (1:200 Cat#A0423, Beyotime, China). The cell nuclei were stained with DAPI (Cat#C1005, Beyotime, China) for 3min at room temperature. A fluorescence microscope (Olympus FV500, Japan) was used to evaluate the tissue slides.

### Complementary DNA synthesis and Quantitative real-time PCR

Total RNA was extracted from lung tissue using Trizol (Takara Bio, Shiga, Japan) according to the manufacturer’s protocol. The quantity and quality of extracted RNA were determined using NanoDrop2000 (Thermo Scientific, USA) at wavelengths of 260 and 280 nm. Purified RNA (1 µg) was treated with DNase, and then reverse transcribed with RevertAid First Strand cDNA Synthesis Kit (Thermo Fisher Scientific, Inc.) according to the manufacturer’s protocol. qPCR was performed using Applied Biosystems 7300 Real-Time PCR System (Applied Biosystems, Branchburg, NJ, USA). The rat-specific primers for *Gal-3 and GAPDH* and human-specific primers for* Zeb-1, Snail, Slug, Twist IL-6, IL-8, NFκB, andβ-actin* (Shenggong Biotech, Shanghai, China) were designed using sequence information from the NCBI database, the primers for real-time PCR were listed in Supplementary Table 2, each sample was analyzed in triplicate, and the levels of cDNA product were normalized to the *GAPDH* gene as the endogenous control.

**Figure 1.**
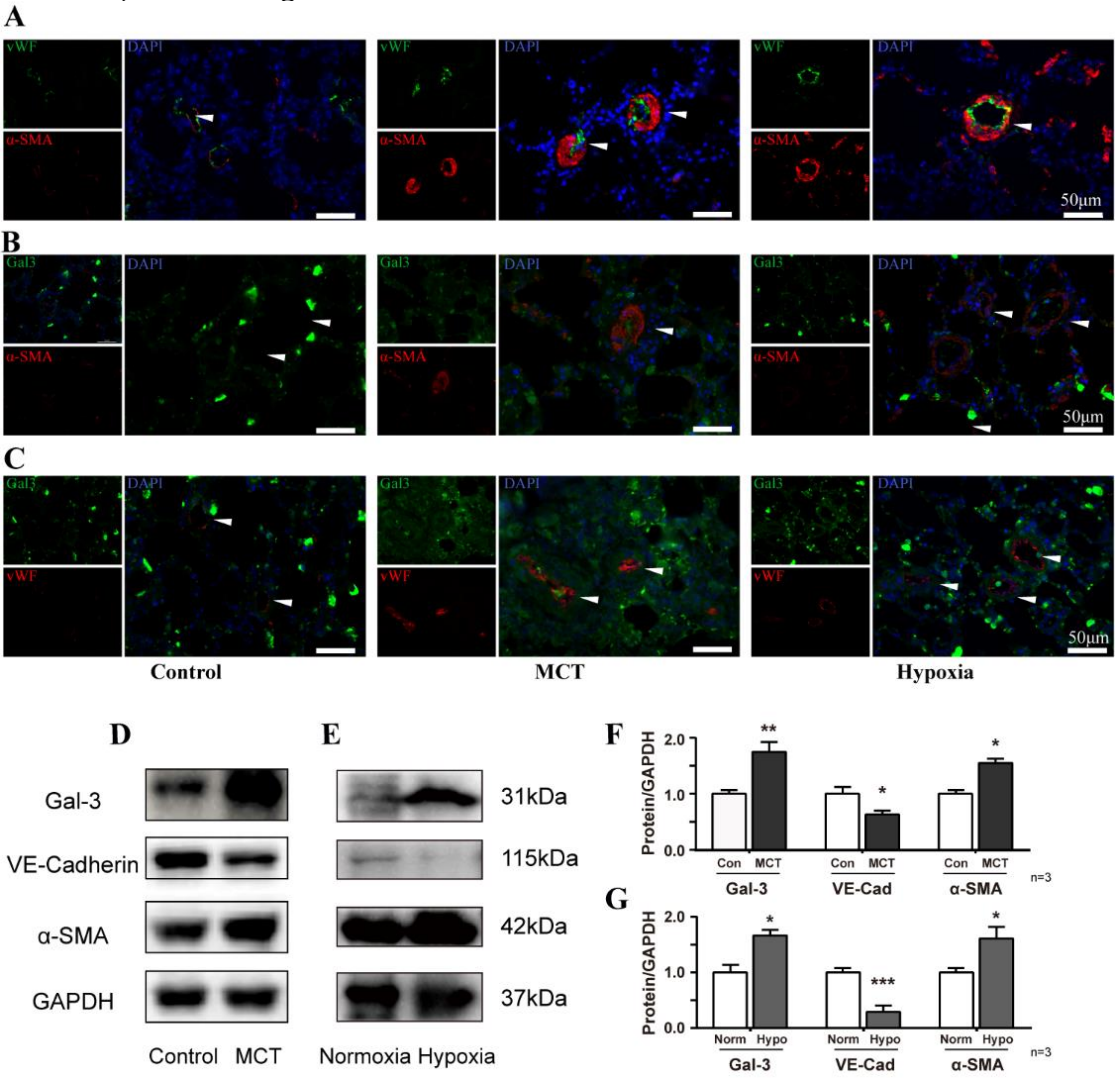
Pulmonary medial and intimal thickness in PAH model, with intimal lesion and endothelial cells migration, Gal-3 localized in medial and neointimal cells, Gal-3 upregulation was consistent with αSMA in model (**A**) Immunofluorescence staining of vWF and αSMA, In pulmonary tissue from control rats, MCT and hypoxia model. Scale bar, 50 μm. (B, C) Immunofluorescence staining of Gal-3 colocalized with vWF and αSMA. Scale bar, 50 μm. (D-E) Immunoblot of lung tissue lysate of MCT and hypoxia treated rat and (F-G) densitometric quantification, n=3. Arrow indicates representative PAs. vWF, von Willebrand Factor. MCT, monocrotaline.

**Figure 2.**
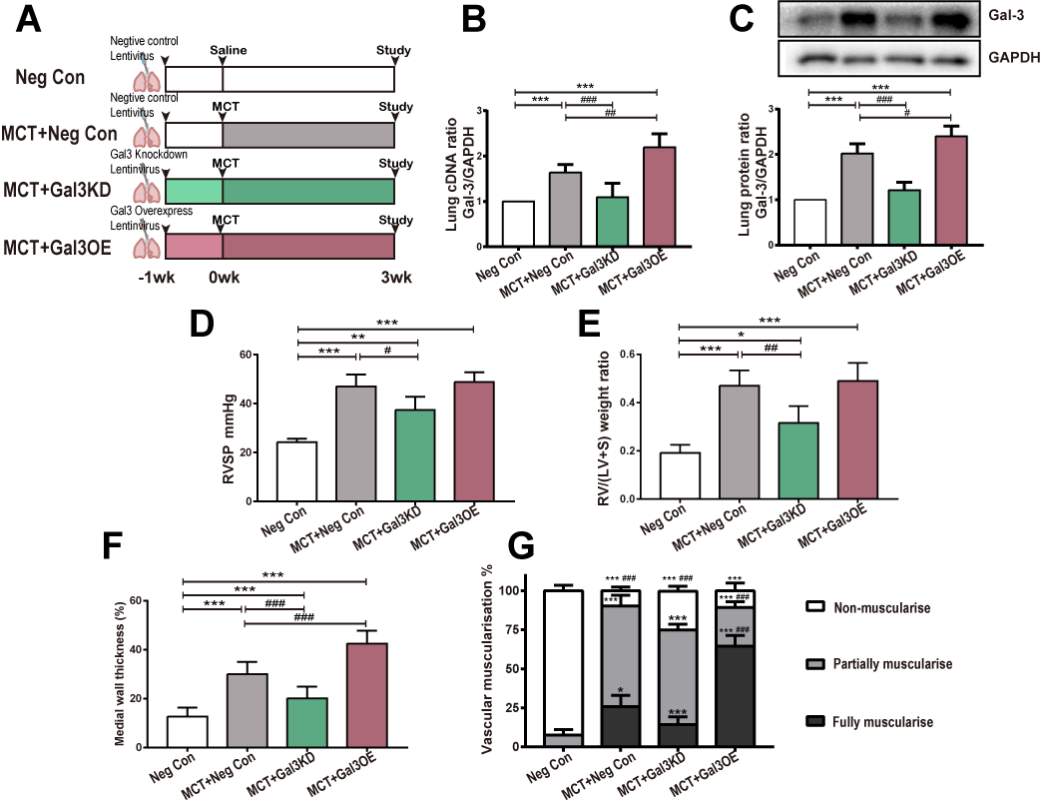
MCT-induced Experimental protocols and PAH phenotype evaluation (**A**) PAH models were established as described (n=5). Seven days after intratracheal delivery of negative control (Neg Con), Gal-3 knockdown (Gal3KD) or Gal-3 overexpression (Gal3OE) lentivirus, Saline or MCT were injected to indicated groups. Three weeks after, rats were studied. (**B**) cDNA and (C) representative immunoblot and relative densitometric of Gal-3 were analyzed. GAPDH was used as loading control. (**D**) Right ventricular systolic pressure (RVSP), (E) right ventricular hypertrophy (right ventricular/ (left ventricular + septum) (RV/LV+S)), (F) medial wall thickness. Muscularization of pulmonary arteries (G), percentage of non- or partially or fully muscularized arteries at alveolar wall and duct level. ^*, **,^ and ^***^ indicate *P* < 0.05, p < 0.01, and p < 0.001, respectively, comparing Neg Con; ^#, ##,^ and ^###^indicate *P* < 0.05, p < 0.01, and p < 0.001, respectively, comparing MCT+ Neg Con. Analyses performed by one-way ANOVA and Bonferroni post hoc. Gal-3, Galectin-3; Neg Con, Negative control; Gal3KD, Gal-3 knockdown; Gal3OE, Gal-3 overexpression.

### Western blotting and quantification

After the indicated duration of treatment, lung tissue samples and cells were homogenized in lysis buffer containing Roche complete protease inhibitor cocktail (Roche, Basel, Switzerland). Concentration normalized protein samples were prepared with SDS loading buffer. 20-30 µg total protein were separated on 12% polyacrylamide gels and transferred onto polyvinylidene fluoride membranes. Membranes were then blocked and probed with one of the following primary antibodies: anti-VE-Cadherin (1:1,000, Cat#2500, Cell Signaling Technology, USA), anti-TGFβ1 (1:1,000, Cat#ab92486, Abcam, UK), anti-αSMA (1: 1,000, Cat#17521-1-AP, Proteintech, USA), anti-Galectin3(1: 1,000, Cat#14979-1-AP, Proteintech, USA), anti-Zeb1(1: 1,000, Cat#3396, Cell Signaling Technology, USA), anti-Slug (1: 1,000, Cat#9585, Cell Signaling Technology, USA), anti-Snail (1: 1,000, Cat#3879, Cell Signaling Technology, USA), anti-twist (1: 1,000, Cat#46702, Cell Signaling Technology, USA), anti-PCNA (1: 2,000, Cat#10205-2-AP, Proteintech, USA), anti-Bax (1: 2,000, Cat#50599-2-IG, Proteintech, USA), anti-Bcl2 (1: 1,000, Cat#12789-2-AP, Proteintech, USA),As a loading control, all blots were reprobed with an antibody toward either anti-GAPDH (1: 1,000, Cat#97166, Cell Signaling Technology, USA) or anti-α-Tubulin (1: 1,000, Cat#2144, Cell Signaling Technology, USA). Densitometry analysis was performed using ImageJ software.

### Statistical analysis

Student’s t-tests were used for comparisons between two groups. Multiple comparisons were assessed by one-way ANOVA, followed by the appropriate post-hoc test for significance, all statistical tests used two-sided tests of significance. All data are reported as mean ± SD. P<0.05 was considered statistically significant. Data analysis was performed using SPSS 20 (IBM SPSS Inc, Chicago, USA) and figures were prepared using GraphPad Prism 6.0 software.

**Figure 3.**
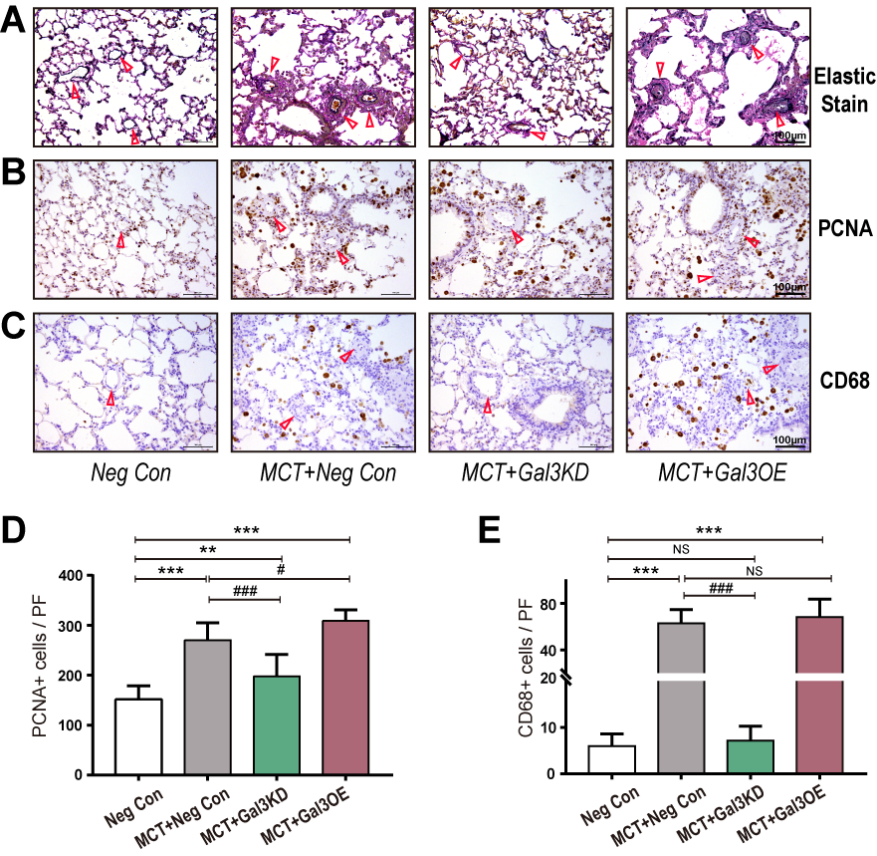
Gal-3 knockdown inhibits MCT induced vascular remodeling and Figure 3 Gal-3 knockdown suppress cell proliferation and macrophage infiltration (**A-C**) Elastic Van Gieson staining and immunostaining in lung tissue from Neg Con, MCT+ Neg Con, MCT+Gal3KD and MCT+Gal3OE group (A) Elastic Van Gieson staining staining to evaluate vasculature occlusive lesions and medial wall thickness of each group. (**B**) PCNA and (C) CD68 to assess proliferation of vessel cells and macrophages related inflammation separately. Arrow indicates representative PAs. (D and E) are semi-quantitative of PCNA (B) and CD68 (D) positive cells in high resolution fields of view (n=30), scale bars, 100μm. ^***^ indicate *P*< 0.001, comparing Neg Con; ^###^ indicate *P*<0.001, comparing MCT+Neg Con. Analyses performed by one-way ANOVA and Bonferroni post hoc. PF: per field.

**Figure 4.**
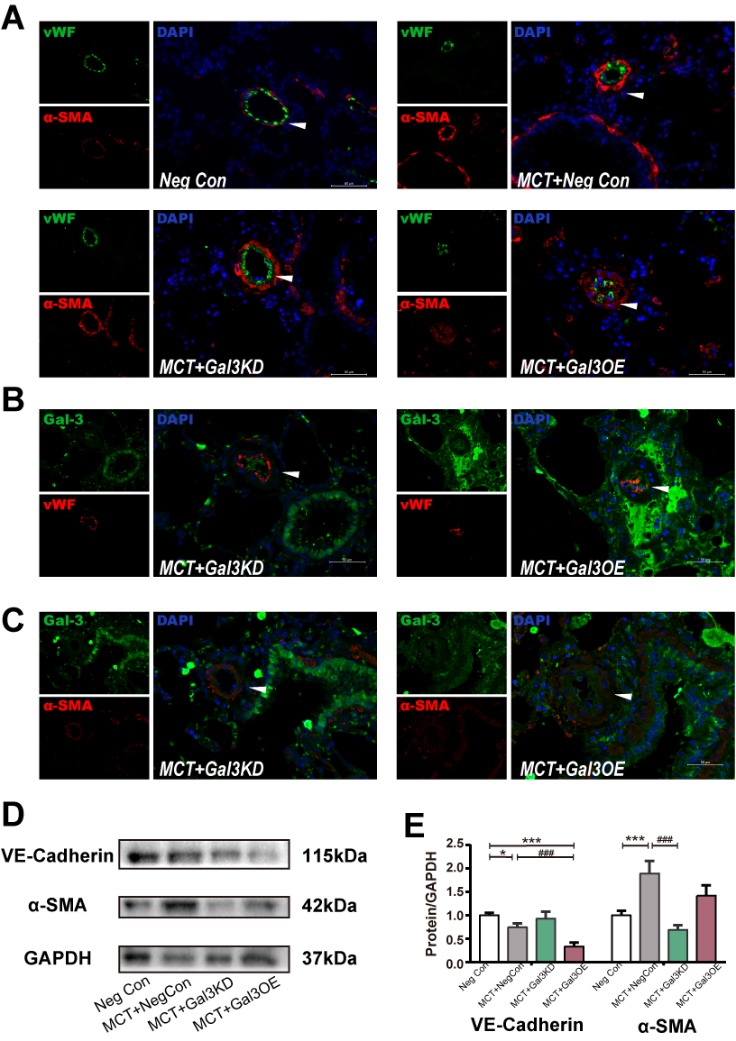
Gal-3 Knockdown can alleviate MCT related vascular hypertrophy through inhibition of EndoMT Representative immunofluorescence images of vWF and αSMA in pulmonary arteries (PAs) tissue sections from Neg Con, MCT+ Neg Con, MCT+Gal3KD or MCT+Gal3OE Rats, Scale bar, 50 μm. (**B** and **C**) Immunofluorescence staining of Gal-3 colocalized with vWF and αSMA. Scale bar, 50 μm. (**D**) Immunoblot of lung tissue lysate of indicated treated rats and (E) densitometric quantification, n=5. Arrow indicates representative PAs. DAPI indicates 4′,6-diamidino-2-phenylindole; Gal-3, Galectin-3; EC, endothelial cell; EndoMT, endothelial-to-mesenchymal transition; Neg Con, Negative control; Gal3KD, Gal-3 knockdown; Gal3OE, Gal-3 overexpression; αSMA, α smooth muscle actin.

**Figure 5.**
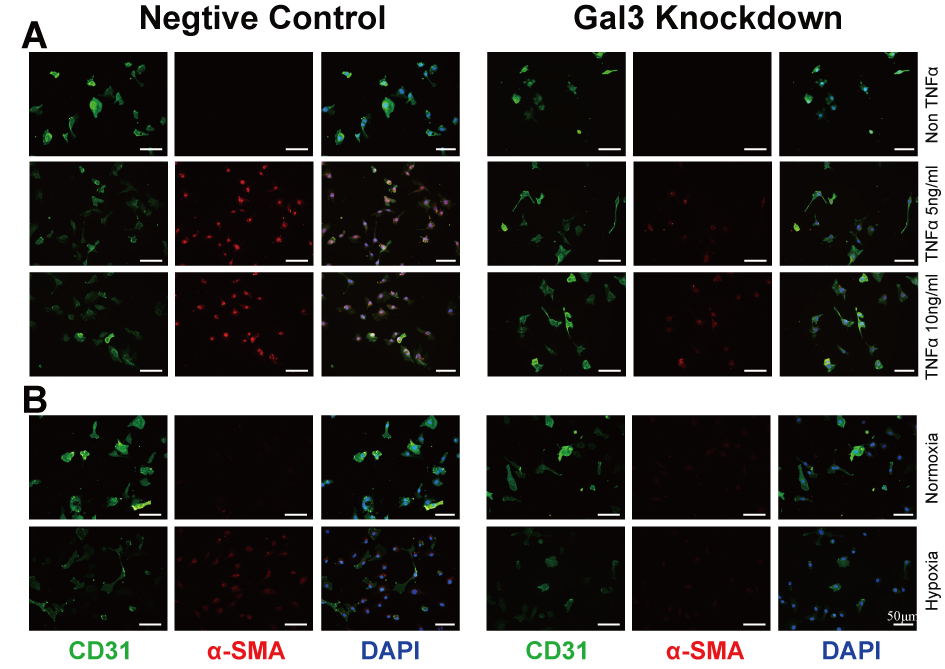
Gal-3 siRNA reduced inflammation and hypoxia caused endothelial cell transition with expression of mesenchymal markers (**A** and **B**) PACE immunofluorescence stained following 48h culture with or without TNFα (5 ng/ml or 10 ng/ml) or hypoxia (O_2_ density 0.5%). (**A**) A dose dependent enhancement of αSMA. (**B**) Suggest hypoxia upregulates αSMA expression. Representative images by microscopy of pulmonary ECs for CD31 (green) and αSMA (red). Nuclei stained with DAPI (blue). Scale bar=50 μm. TNFα, tumor necrosis factor α; DAPI indicates 4′,6-diamidino-2-phenylindole; EC, endothelial cell; PAEC, pulmonary arterial endothelial cell; αSMA, α smooth muscle actin.

## RESULTS

### Smooth muscle cells proliferation and neointimal hyperplasia, accompanied with Gal-3 upregulation in remodeled vessels of PAH animal model

We observed profound vascular remodeling in MCT and hypoxia rat model. SD rats fed in normoxia and saline injection were taken as control group in Figure 1A, Single thin layer of ECs expressing vWF at EC to EC junctions immediately adjacent to smooth muscle cells, However, αSMA and vWF were increased in PAH model compared with control group, and PAH model vessel ECs layer dissociated, some of lumen cells was co-express of vWF and αSMA, accompanied part of endothelial cells migrate into inner layer of the vessel.

Both thickening of intimal and increased smooth muscle cells were contributed in reduction of peripheral arterial volume. Gal-3 was shown to co-express with αSMA and vWF and accompanied with their upregulation (Fig. 1B, C). In addition, we found Gal-3 and αSMA upregulated in PAH model and decreased level of VE-Cadherin (Fig. 1D, E). Besides, MCT induced model suffered more severe vascular hypertrophy and endothelial layer migration, which imply MCT model underwent significant vessel remodeling and it is better for understanding of the underline pathology of endothelial mesenchymal transition in PAH.

### Reduction of Gal-3 reverses MCT-induced PAH with attenuated hemodynamic parameters, right ventricular hypertrophy and pulmonary arteries remodeling.

For more profound vascular abnormalities observed in MCT model, our study applied Gal-3 knockdown and overexpression lentivirus to MCT rats rather than hypoxia model to further investigate the importance of Gal-3 in regulating EndoMT in PAH. Intratracheal lentivirus Neg Con, Gal3KD and Gal3OE were administered in SD rats. Animal models were established as shown in Figure 2A. Evaluation by qPCR and immunoblotting showed that MCT induced Gal-3 upregulation was suppressed following Gal3KD. The MCT+Gal3OE group showed an even higher Gal-3 expression in cDNA and protein level than MCT+ Neg Con group (Fig. 2B, C). Pulmonary hemodynamic and right heart remodeling assessment showed that the MCT-treated group developed exaggerated right ventricular systolic pressure (RVSP) and weight ratio of right ventricular/(left ventricular + septum) (RV/(LV+S)), and Gal3KD was able to prevent MCT induced right heart hypertrophy. However, RVSP and right heart hypertrophy did not differ between MCT and MCT+Gal3OE groups (Fig. 2D, E). As expected, both RVSP and RV/(LV+S) were significantly higher in the hypoxia group than in the normoxia group (Supplementary Fig. 1).

We have employed lentivirus mentioned above to PAECs (MOI 20 for 72h seen in Supplementary Fig. 2) found that virus could interfere Gal-3's expression, indicating that lentivirus may have essential effect in vascular remodeling. To further explore Gal-3 in pulmonary vascular thickening, quantification of vascular muscularization and medial wall thickness of each group were performed (Fig. 2F, G). Compared with the Neg Con group, fully and partially muscularized pulmonary vasculature as well as medial wall thickness was significantly greater in the MCT group. When compared to MCT rats, the percentage of non-muscularized tissue was increased in the MCT+Gal3KD group. A similar amount of non-muscularized vessels, but more occlusive lesions and less partially muscularized pulmonary vasculature was seen in MCT+Gal3OE rats. According to muscularization quantification, Gal3KD appeared to reverse Pulmonary vascular structural changes by increasing the number of non-muscularized vessels and reducing occlusive lesions. Gal3OE considerably enhanced obliterated vessels (Fig. 3A), which is commonly associated with increased PA resistance in PAH.

**Figure 6.**
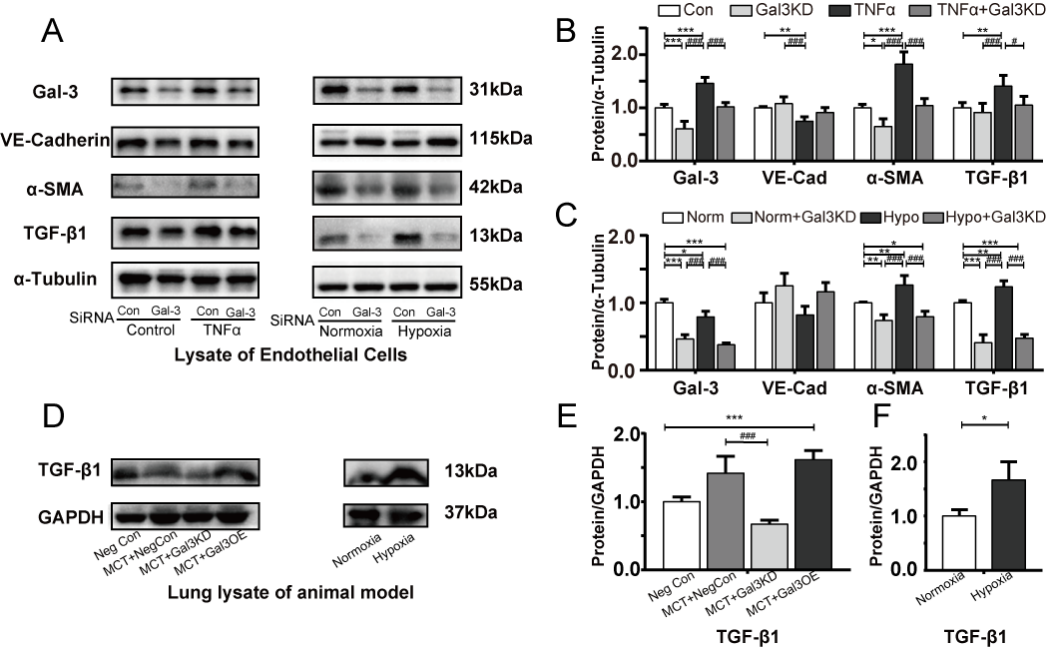
Gal-3 inhibition reverses EndoMT in these models Representative immunoblots and relative densitometric analysis of EndoMT relative proteins. (A, C) hPAECs transfected with Non-targeting (Con) siRNA or Gal-3 SiRNA and exposed to TNFα (10 ng/ml) for 48 hours compared with controls (n=5). (B, D) hPAECs transfected Con and Gal-3 SiRNA followed by normoxia (21% O_2_) or hypoxia (0.5% O_2_) for 48 h (n=5). and (E, G) whole lung lysates of Neg Con, MCT+ Neg Con, MCT+Gal3KD, MCT+Gal3OE PAH model (n=5). (F, H) Immunoblots and quantification of lung lysates of normoxia (21% O_2_) and hypoxia rats (10% O_2_), GAPDH and α-Tubulin were used as loading control. Bars represent mean ± SEM. ^*, **,^ and ^***^ indicate p < 0.05, p < 0.01, and p < 0.001, respectively, comparing Control group (Neg Con lung, Con PAEC, Normoxia rat lung or Normoxia PAEC); ^#, ##,^ and ^###^ indicate p < 0.05, p < 0.01, and p<0.001, respectively, comparing the PAH related stimuli (MCT+ Neg Con rat lung, PAEC after TNFα, hypoxia lung or hypoxia PAEC. Gal-3, Galectin-3; PH, pulmonary hypertension; MCT, monocrotaline; TNFα, tumor necrosis factor α; EndoMT Endothelial-mesenchymal transition; α-SMA, α smooth muscle actin; PA, pulmonary artery; PAEC pulmonary artery endothelial cell; Norm, Normoxia; Hypo, Hypoxia; Student’s t-test was used for comparing differences in normoxia and hypoxia groups. Analyses were performed by one-way ANOVA and Bonferroni post hoc.

### Reversal of pulmonary vascular proliferation and perivascular inflammation infiltration by reduced Gal-3

Ki67 expression in PAs was increased in the MCT-treated groups and was down-regulated following Gal3KD pretreatment (Fig. 3B, D). The MCT+Gal3OE group showed dramatically enhanced perivascular Ki67 expression. Prominent infiltration of macrophages (CD68^+^)[19] with a preferential accumulation surrounding the peripherally remodeled vessels was also noted in MCT rats. CD68 was markedly suppressed by Gal3KD treatment (Fig. 3C, E).

### Reduced Gal-3 reverses MCT-induced PAH through downregulation of EndoMT in intimal lesions

The MCT model exhibits familiar pathologic vascular changes often observed in clinical. From the results above, Gal-3 appears to enhance the development of PAH through pulmonary vascular remodeling. To further characterize the relationship between Gal-3 expression and EndoMT, we studied the MCT induced PAH model with or without Gal-3 intervention (knockdown or overexpression) by intratracheal lentivirus mentioned above and sought to determine the presence of EndoMT in an *in vivo* PAH model. EndoMT was determined by colocalization of vWF and the mesenchymal marker αSMA. In addition, Gal-3/vWF and Gal-3/αSMA colocalization were applied to assess to illustrate the role of Gal-3 in EndoMT.

The MCT group showed a distinct increase in expression of αSMA in vascular lumen compare to the Neg Con Group (Fig. 4A). In control pulmonary arteries, we observed a single thin and integrated layer of ECs expressing vWF, which was immediately adjacent to smooth muscle cells expressing αSMA. In intimal lesions from MCT rat lungs, we observed a swollen luminal layer of cells expressing endothelial markers. In addition, αSMA cells exhibited excessive proliferation in MCT rats with hypertrophy pulmonary vascular. Figure 4B and 4C revealed Gal3KD decrease Gal-3 expression in intima and media wall with significant reverse of remodeled vessels. However, Gal3OE upregulated Gal-3 and resulted in more profound vascular narrowing and even obliteration.

To evaluate the role of Gal-3 in EndoMT of MCT lungs, morphological analysis was performed using MCT+Gal3KD and MCT+Gal3OE models (Fig. 4B, C). Compared to MCT rats, the MCT+Gal3KD group showed less advanced vessel narrowing, a complete intimal layer and reduced vascular occlusive lesions. However, in MCT+Gal3OE rats, changes associated with PAH including occlusive lesions were enhanced. We observed mixture of ECs and smooth muscle stained by vWF and αSMA respectively. Pulmonary vascular remodelling characterized by αSMA positive cells over-proliferation and ECs dissociate from monolayer of tightly cohesive cells at the abluminal surface of the vessel and migrate to inner tissue, and diffusion staining of ECs and SMCs could be observed in hypertrophy vessel. We also observed accompanied with Gal-3 knockdown or overexpression, endothelial and mesenchymal marker dysregulated in models. Gal3KD prevented αSMA further elevation. In contrast, VE-Cadherin met a significant reduction in Gal3OE PAH model (Fig. 4D, E).

Gal3KD showed less occlusive intimal lesion, and Gal3OE exacerbated vessel stenosis. These findings imply that Gal-3 plays an important role in PAH, at least in part, by affecting EndoMT related to PA remodeling.

### Reduced Gal-3 reverses inflammation induced EndoMT in vitro

To explore whether Gal-3 was involved in EndoMT *in vitro*, we utilized two different cell models: the TNFα model and the hypoxia model, used to mimic inflammation and hypoxia model separately. We used commercial PAECs transfected with Gal-3 targeted siRNA to evaluate whether EndoMT occurs under TNFα or hypoxia stimuli. We also evaluated whether EndoMT could be reversed as previously shown in *in vivo* experiments.

Significantly reduced αSMA was seen in PAECs transfected with Gal-3 siRNA as visualized by immunofluorescent staining (Fig. 5). TNFα (5 ng/ml and 10 ng/ml respectively) and hypoxia treated cells showed increased αSMA, which was reduced after knockdown of Gal-3. These results were consistent with in *in vivo* experiments described above, where Gal3KD reversed EndoMT. In addition, increased Gal-3 was seen after TNFα treatment (supported by Supplemental Fig. 3 Immunofluorescence and western blot seen in Figure 6A, C). Interestingly, and unlike our *in vivo* experiments, 48 hours of hypoxia did not increase, but decreased Gal-3 expression in PAECs. αSMA expression was also less apparent. It may because of Gal-3 underwent other protective down-regulation in early phase in hypoxia in endothelial cells. We also assessed whether the increase in TNFα with depletion of Gal-3 in PAECs could be mediating a downregulation in mRNA expression of the pro-inflammatory cytokines, interleukin 6 (IL-6), IL-8 and NFκB (Supplmentary Fig. 4). We found Gal-3 siRNA could inhibit IL-8 elevation, IL-6 and NFκB were not evidently suppressed with Gal-3 inhibition.

**Figure 7.**
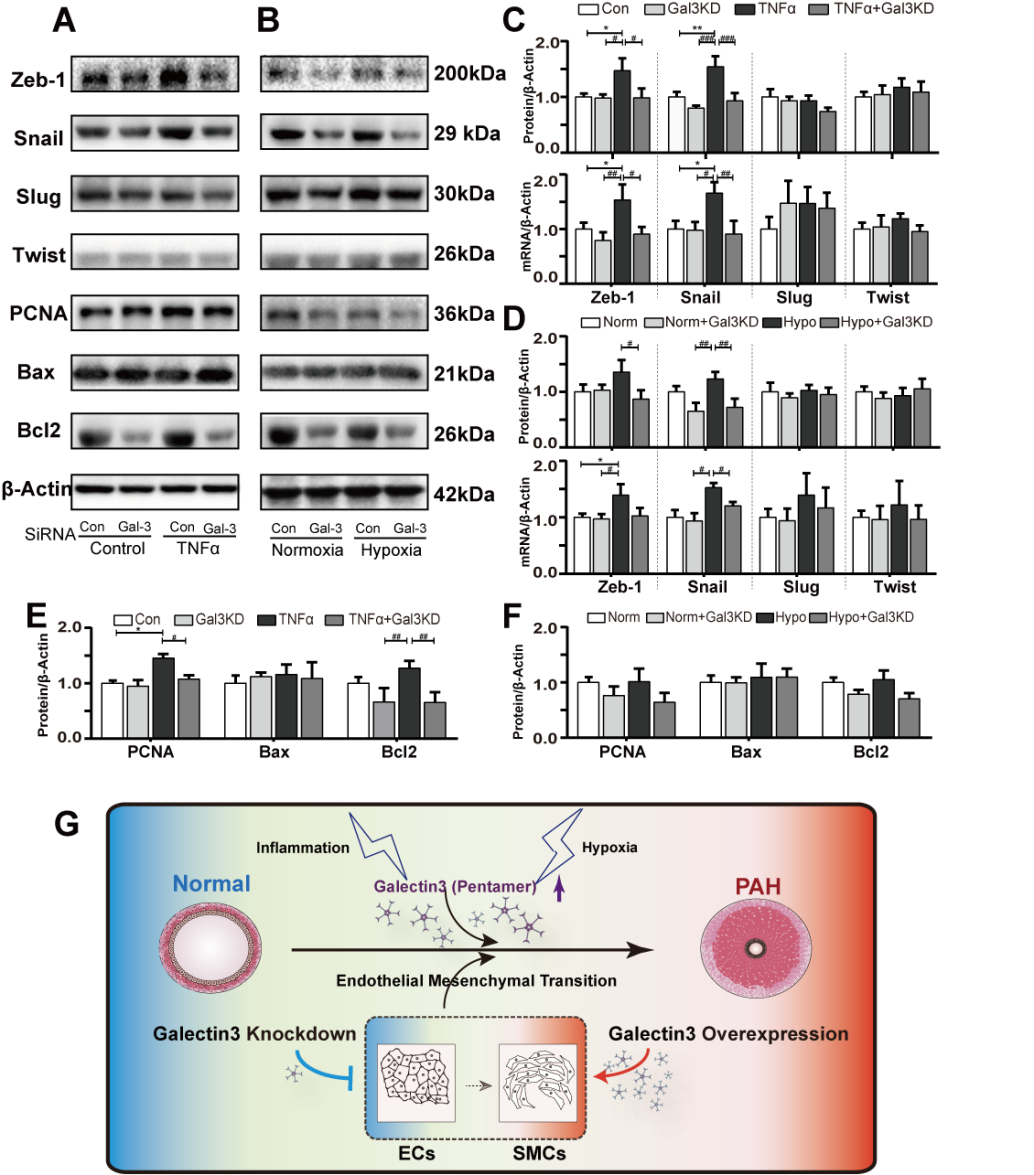
Gal-3 inhibition partially alleviate EndoMT associated transcription factor activation and hyper-proliferation, anti-apoptosis marker upregulation Representative immunoblots and relative densitometric analysis of EndoMT associated transcription factor and proliferation, apoptosis marker. (**A**) human PAECs transfected with Non-targeting (Con) siRNA or Gal-3 SiRNA and exposed to TNFα (10 ng/ml) for 48 hours compared with controls (n=3). (**B**) PAECs transfected Con and Gal-3 SiRNA followed by normoxia (21% O_2_) or hypoxia (0.5% O_2_) for 48 hours. (C, D) qRT-PCR and Immunoblots quantification of EndoMT related transcription factor (E, F) Immunoblots quantification of proliferation, apoptosis and anti-apoptosis marker, β-actin were used as loading control. Bars represent mean ± SEM. ^*, **,^ and ^***^ indicate p < 0.05, p < 0.01, and p < 0.001, respectively, comparing Control group; ^#, ##,^ and ^###^ indicate p < 0.05, p < 0.01, and p < 0.001, respectively, comparing the TNFα (A, C, E) or hypoxia (B, D, F) PAEC. Student’s t-test was used for comparing differences in normoxia and hypoxia groups. Analyses performed by one-way ANOVA and Bonferroni post hoc. (**G**) A schematic model showing the proposed mechanism of Gal-3 regulation. Exposure to PAH related stimuli (hypoxia and inflammation). In the presence of these factor (MCT and hypoxia) and cytokine (TNFα), Gal-3 is upregulated leading to pulmonary vascular remodeling and development of PAH, EndoMT is involved in this pathological process

### Gal-3 intervention induces endothelial/mesenchymal marker protein changes and regulates proliferation/ apoptosis balance

To quantify the extent of EndoMT in each PAH model *in vitro*, and to evaluate the significance of Gal-3 in EndoMT and its relative markers, VE-cadherin, αSMA and TGFβ1 (predominant profibrotic signal in EndoMT) were assessed in each cell model.

As a result of elevated Gal-3 previously shown, TNFα leads to increased smooth muscle markers and reduced endothelial markers (Fig. 6). Gal-3 siRNA was able to inhibit TNFα induced αSMA and TGFβ1 upregulation. For hypoxia endothelial cells, Gal-3 knockdown could inhibit hypoxia induced αSMA and TGFβ1 elevation in hPAECs VE-cadherin expression was not enhanced significantly when pretreatment with Gal-3 siRNA. As described above in EC immunofluorescent staining, Gal-3 was down-regulated in response to hypoxia. This may be due to some unknown early protective mechanisms.

We observed the mRNA and protein expression of Zeb-1, Snail, Slug and twist, which are critical transcription factor of EndoMT induction (Fig. 7A-D). Zeb-1 and snail were increased with the administration of TNFα and hypoxia. The expression of Slug and twist was not significantly regulated by Gal-3 knockdown.

Proliferation and apoptosis index were analyzed in TNFα and hypoxia treated hPAEC. As shown in Fig. 7A, B, E and F, PAECs pretreated with Gal-3 siRNA restore PCNA and Bcl2 elevation induced by TNFα. Bax did not met significantly change in each group. in hypoxia treated cells, PNCA, Bcl2 and Bax expression were not distinct in each group.

## DISCUSSION

The present study identifies a novel role for Galectin-3 in promoting EndoMT in PAH. We show that EndoMT occurs in experimental PAH induced by MCT or hypoxia, and Gal-3 is highly expressed in PAH PA lesions. Moreover, after Gal-3 is upregulated by Gal3OE lentivirus in an MCT model, more severe intimal lesions were observed. On the contrary, targeted Gal-3 lentivirus delivery alleviated right ventricular systolic pressure and reduced vascular abnormalities. Gal3KD prevented vascular cell over-proliferation and macrophage infiltration. Gal-3 may activate the pro-fibrotic process through TGFβ1. Consequently, we have shown that ECs are a source for αSMA-positive cells that build up in PAH vascular lesions, and that alteration in the Gal-3 could be a possible cause of EndoMT. Collectively, we suggest that the Gal-3 may be an attractive target for the prevention and treatment of severe PAH models. These beneficial effects can be explained, at least in part, by inhibition of EndoMT in pulmonary vasculature.

Pulmonary arterial hypertension is characterized by enhanced αSMA expressing cell proliferation and inflammation[20], which contribute to increased pulmonary artery wall thickness and resistance. In the present study, Gal3KD pretreated MCT rats showed reduced vascular wall thickness and macrophage infiltration. In addition, Gal3OE enhanced proliferation of αSMA positive cells in MCT rats. Gal-3 is a protein member of β-galactoside binding family[8], and the expression of this lectin has been reported in fibroblasts[21], endothelial cells[22] and inflammatory cells[23]. Previous studies have demonstrated Gal-3 is involved in fibrotic diseases involving the heart[21] and kidney[24], which ultimately leads to organ remodeling and dysfunction. Pro-fibrotic and pro-inflammatory ability are both recognized as mediators of pulmonary vascular change in PAH[25]. Increasing number of studies have focus their attention in Gal-3 and PAH in recent years. Barman[26] observed time dependent increase in Gal-3 expression in isolated pulmonary arteries of rat. Then 2 kinds of Gal-3 inhibitor and Gal-3 genetic knockout rats were employed to established PAH model, determined Gal-3 inhibition ameliorate PAH. Hao[27] demonstrated Gal-3 inhibition reduces increased RVSP and right ventricular hypertrophy of mice in hypoxia. Besides, Gal-3 reduction could alleviate HPASMC proliferation in vitro. Mazurek[28] measured Gal-3 level in PH, PAH and heart failure with preserved ejection fraction associated PH to assess prognostic value of Gal-3, demonstrated Gal-3 is a strong, independent prognostic marker in PH. Through these findings were helpful to understand Gal-3 is evident to PH, how Gal-3 contribute to PAH vessel occlusive lesions and increase right heart load is still opaque, only when more comprehensive understand the mechanism of Gal-3 in vascular remodeling can we find a precise and attractive target for treatment to PAH.

Pro-fibrotic and pro-inflammatory pathways are also key mediators of EMT[29, 30], and Gal-3 has been demonstrated to be involved in the EMT process. MacKinnon et al.[14] have illustrated that Gal-3 regulates TGF-β1 driven lung fibrosis. TGF-β isforms elevation has been found in pulmonary arteries from patients with IPAH[31] and MCT model[32, 33], recent finding revealed BMPR2 and TGF-β signaling pathway complex crosstalk play significant role in PAH pathogenesis[5, 32, 33], TGF-β1 is principal growth factor responsible for organ fibrosis. Similar to MacKinnon’s study[14], we have revealed that Gal-3 knockdown could partially reverse EndoMT in accordance with TGF-β1 downregulation. Interestingly, Wang [34]have recently demonstrated Gal-3 is required for TGF-β1 stimulation via STAT3 signaling, take our study into account, Gal-3 may have interaction with TGF-β1 which need further experiment to confirm, (eg. Gal-3 and TGF-β1 directly interaction could be verified by co-immunoprecipitation) EndoMT and EMT share many common features[35] and there are likely triggers and pathways that occur similarly between both processes. Both EndoMT and EMT result in enhanced expression of mesenchymal markers. Gal-3 has been proven to play a role in EMT related diseases and may also act to regulate EndoMT.

We previously found that exogenous Gal-3 recombinant protein stimulated pulmonary artery fibroblast and cardiac fibroblast proliferation and collagen deposition. Furthermore, serum Gal-3 levels were significantly elevated in human PAH patients.[15, 16] These results support previous observations showing that Gal-3 blockade may be protective against hypertrophy and remodeling, and strengthen the hypothesis that Gal-3 targeted therapies may be useful in the treatment of pulmonary arterial hypertension.

We demonstrate a novel relationship in PA endothelial cells whereby Gal-3 and TGFβ1 are associated with increased expression of the mesenchymal marker αSMA. Figure 1 revealed that in PAH model ECs layer dissociated, vWF and αSMA co-expressed in some of EC cells, with part of ECs migrate toward to inner layer, which indicate that endothelial mesenchymal transition occurs in MCT and hypoxia PAH models, in addition, Figure 1B and 1C shown Gal-3 co-expressed with αSMA and vWF and accompanied with their upregulation suggesting that Gal-3 may regulate endothelial cells transition in vascular pathologies.

Following exposure to inflammatory and hypoxic stimuli, Gal-3 was upregulated in accordance with αSMA. In support of these protein changes in animal models, we found Gal-3 knockdown could restore hemodynamic and right heart hypertrophy caused by MCT, meanwhile, Gal-3 overexpression did not aggravate right ventricular systolic pressure and RV/(LV+S) weight ratio noteworthily (Fig. 2D and 2E). At the same time, Gal3kd alleviated MCT-induced Pulmonary vascular structural changes by reduced media wall thickness and increase non-muscularized percent of vessels, Gal3OE dramatically increased medial wall thickness as well as percent of occlusion lesion vassels (Fig. 2F and 2G) which suggesting Gal-3 knockdown could prevent PAH development by reversed vascular change and inhibit followed hemodynamic disorder and right ventricular hypertrophy. Gal-3 overexpression enhanced vessel media muscularization but did not promote right ventricular weight ratio and RVSP, implying that Gal-3 overexpression would bring to profound vassels narrowing rather than hemodynamic change in these 21 days observation time window. Given more time for study of PAH development of MCT + Gal3OE rats, maybe subsequent RVSP and Fulton index deterioration will be revealed.

Vascular cell proliferation is one of the paradigm of PAH, the increasing number of cells expressing αSMA has been traditionally thought closely related to the proliferative expansion of resident vascular media SMC[4]. EndoMT was regulated by inflammatory signaling, and PAH is also consistently characterized by early and persistent in inflammation. The inflammatory microenvironment observed in PAH contains mounts of signaling proteins, such as TNFα, interleukin-1β, interleukin-6 and reactive oxygen species. These inflammatory factors may have individual effects on EndoMT process. In Figure 3, we investigated the effect of Gal-3 intervention in proliferation and inflammation of MCT rats, evaluated by Ki67 and CD68 staining. In our study, we found Gal3KD pretreated rats were protected from pro-inflammatory and over-proliferation effect of MCT, meanwhile, Gal3OE rats underwent excessive proliferation. These results support Gal3KD could alleviate vascular remodeling shown in Figure 1.

In Figure 4, when we applied Gal3OE and Gal3KD lentivirus models to MCT rats, where we found that Gal3OE could promote PA occlusive lesions, break down the intimal single layer structure, and upregulate Gal-3 and αSMA positive cells. In addition, in the Gal3KD group, lesion thickness was reduced. Disruption of the endothelial cell layer is an early phenomenon in PAH[20]. The high expression of Gal-3 and αSMA in endothelial cells might suggest that, increased Gal-3 can aggravate EndoMT process and promote the extend of vascular structure changes in PAH. Results in Figure 4 further support that the role of Gal-3 in mediating vascular remodeling.

Transfection of siRNAs targeting Gal-3 prevented the increase in αSMA at the mRNA and protein level. The repression of αSMA by Gal-3 siRNA in the context of TNFα and hypoxia were consistent with these findings. EndoMT was partially ameliorated by silencing Gal-3 signaling. Surprisingly, hypoxic endothelial cells underwent a reduction in Gal-3 expression after 48h. It may be that Gal-3 is down-regulated by other protective factors in hypoxia, however this is not currently clear and requires further investigation.

For further determination of Gal-3 in regulating EndoMT. associated transcription factor, such as Twist, Snail, Slug, Zeb-1 were needed to investigate, we found Gal-3 siRNA suppressed the elevation of Zeb-1 and Snail in TNFα and hypoxia treated hPAECs. Which indicate Gal-3 may mediate EndoMT through Zeb-1 and Snail, rather than slug or twist.

Accumulated evidences indicated that EndoMT is associated with over proliferation, anti-apoptosis in diverse etiologies. At the same time, excessive proliferation and resistance to apoptosis of the vascular cells is the characteristic feature of PAH. So, we detected proliferation pro-apoptosis and anti-apoptosis marker in our hPAEC, we found TNFα led to hyper-proliferation and pro-apoptosis upregulation, which is in accordance to pathogenesis of PAH. Furthermore, Gal-3 knockdown abolish PCNA and Bcl2 elevation when combine with TNFα administrated. Bax did not met obviously change. However, we did not observe significant change of PCNA, Bcl2 or Bax in hypoxia induced cells, that may because of hypoxia only induced slightly endothelial cells phenotype change, then over-proliferation and inhibition of apoptosis is not significant.

A limitation of our study results was that we did not apply Gal3OE and Gal3KD lentivirus to a hypoxic rat model, because in our study there only modest vascular remodeling and EndoMT occurred in hypoxia model. A great deal of work remains to illustrate that EndoMT is responsible for the pulmonary vascular structure changes that develops in this model, and to determine what could be the second hit that allows PAH occurrence in the context of Gal-3 alterations.

In Summary, our work exhibits that EndoMT participates in vascular remodeling in PAH, and that Gal-3 inhibition may have therapeutic implications for PAH. The identification of the key molecular players of EndoMT in PAH, and the mechanisms participating in the control of their expression and of their functions requires ongoing research, however EndoMT could be a new target in PAH therapeutics.

## Supplemetary Materials

The Supplementary data can be found online at: www.aginganddisease.org/EN/10.14336/AD.2018.1001


