# Supplementary material for: Galectin-3 Mediates Endothelial-to-Mesenchymal Transition in Pulmonary Arterial Hypertension
Source: Aging Dis. 2019 Aug 1;10(4):731–45. doi: 10.14336/AD.2018.1001 (PMC6675525; doi:10.14336/AD.2018.1001)
Supplement: Supplementary file 1 [file AD-10-4-731-s.pdf]

SUPPLEMENTARY DATA

Galectin-3 Mediates Endothelial-to-Mesenchymal Transition in Pulmonary Arterial Hypertension

Tangzhiming Li[1], Lihuang Zha[1], Hui Luo[1], Suqi Li[2], Lin Zhao[3], Jingni He[1], Xiaohui Li[4], Qiangqiang Qi[1], Yuwei Liu[1], ZaixinYu[1] *

[1]Department of Cardiology, Xiangya Hospital, Central South University, Changsha, China
[2]State Key Laboratory of Cardiovascular Disease, Fu Wai Hospital, National Center for Cardiovascular Diseases, Chinese Academy of Medical Sciences and Peking Union Medical College, Beijing, China
[3]Centre for Pharmacology and Therapeutics, Division of Experimental Medicine, Imperial College London, Hammersmith Hospital, London W12 0NN, UK
[4]Department of Pharmacology, School of Pharmaceutical Sciences, Central South University, Changsha, China

© 2018. Li T et al. Published online at http://www.aginganddisease.org/EN/10.14336/AD.2018.1001

SUPPLEMENTARY DATA

Supplementary Table 1. Gal-3 knockdown and overexpression sequences.

**Gal-3 knockdown sequence (lentivirus)**
CAATCATAGGCACAGTGAA
**Gal-3 overexpression sequence (lentivirus)**
ATGGCAGACAATTTTTCGCTCCATGATGCGTTATCTGGGTCTGGAAACC CAAACCCTCAAGGATGGCCTGGCGCATGGGGGAACCAGCCTGCTGGGG CAGGGGGCTACCCAGGGGCTTCCTATCCTGGGGCCTACCCCGGGCAGG CACCCCCAGGGGCTTATCCTGGACAGGCACCTCCAGGCGCCTACCCTGG AGCACCTGGAGCTTATCCCGGAGCACCTGCACCTGGAGTCTACCCAGG GCCACCCAGCGGCCCTGGGGCCTACCCATCTTCTGGACAGCCAAGTGCC ACCGGAGCCTACCCTGCCACTGGCCCCTATGGCGCCCCTGCTGGGCCAC TGATTGTGCCTTATAACCTGCCTTTGCCTGGGGGAGTGGTGCCTCGCAT GCTGATAACAATTCTGGGCACGGTGAAGCCCAATGCAAACAGAATTGC TTTAGATTTCCAAAGAGGGAATGATGTTGCCTTCCACTTTAACCCACGC TTCAATGAGAACAACAGGAGAGTCATTGTTTGCAATACAAAGCTGGAT AATAACTGGGGAAGGGAAGAAAGACAGTCGGTTTTCCCATTTGAAAGT GGGAAACCATTCAAAATACAAGTACTGGTTGAACCTGACCACTTCAAG GTTGCAGTGAATGATGCTCACTTGTTGCAGTACAATCATCGGGTTAAAA AACTCAATGAAATCAGCAAACTGGGAATTTCTGGTGACATAGACCTCA CCAGTGCTTCATATACCATGATA

© 2018. Li T et al. Published online at http://www.aginganddisease.org/EN/10.14336/AD.2018.1001

SUPPLEMENTARY DATA

Supplementary Table 2. The primers for Quantitative Real-Time PCR were listed as followed

**Galectin 3**	Rat	**Former**	**5’-TGCTACTGGCCCCTTTGGTG-3’**
		Reverse	5’-AAGTGGAAGGCGATGTCGTT-3’
**GAPDH**	Rat	Former	5’-CTCTACCCACGGCAAGTTC -3’
		Reverse	5’-GCCAGTAGACTCCACGACATA-3’
**Slug**	human	Former	5’-CATTAGAACTCACACGGGGA-3’
		Reverse	5’-CGAGTAAACATTGATTGCGTCAC-3’
**Snail**	human	Former	5’-CCCTCAAGATGCACATCCGAA-3’
		Reverse	5’-GGCACTGGTACTTCTTGACAT-3’
**Twist**	human	Former	5’-GGCCGGAGACCTAGATGTCA-3’
		Reverse	5’-ATGCAGAGGTGTGAGGATGGTG-3’
**Zeb-1**	human	Former	5’-GATGATGAATGCGAGTCAGATGC-3’
		Reverse	5’-ACAGCAGTGTCTTGTTGTTGT-3’
**IL-6**	human	Former	5’-GGTACATCCTCGACGGCATC-3’
		Reverse	5’-GTCAGGGGTGGTTATTGCAT-3’
**IL-8**	human	Former	5’-TGTAAACATGACTTCCAAGCTG-3’
		Reverse	5’-TTCACTGATTCTTGGATACCAC-3’
**NF-κB**	human	Former	5’-TGGCAGCTCTTCTCAAAGCA-3’
		Reverse	5’-AGCTGTTTCATGTCTCCTTGTGC-3’
**β -Actin**	human	Former	5’-ACCCAGATCATGTTTGAGACC -3’
		Reverse	5’-TCATGAGGTAGTCAGTCAGGT -3’

© 2018. Li T et al. Published online at http://www.aginganddisease.org/EN/10.14336/AD.2018.1001

SUPPLEMENTARY DATA

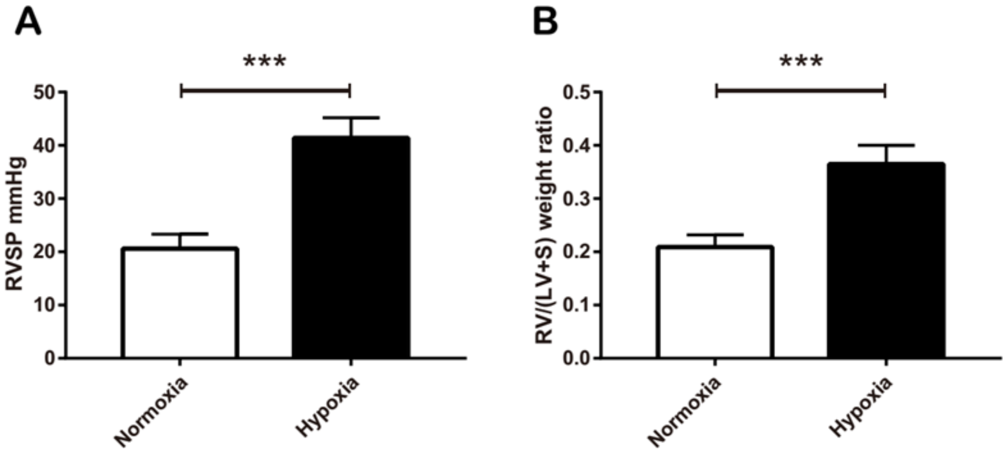

Supplementary Figure 1. Hypoxia leads to rat PAH. (A) Hemodynamic analysis between the normoxia and hypoxia group, (B) right ventricular hypertrophy (right ventricular/ (left ventricular + septum) (RV/LV+S)), ### indicate *P*< 0.001, compare to normoxia. Student's t-tests were used for comparisons of normoxia and hypoxia groups. Analyses performed by one-way ANOVA and Bonferroni post hoc.

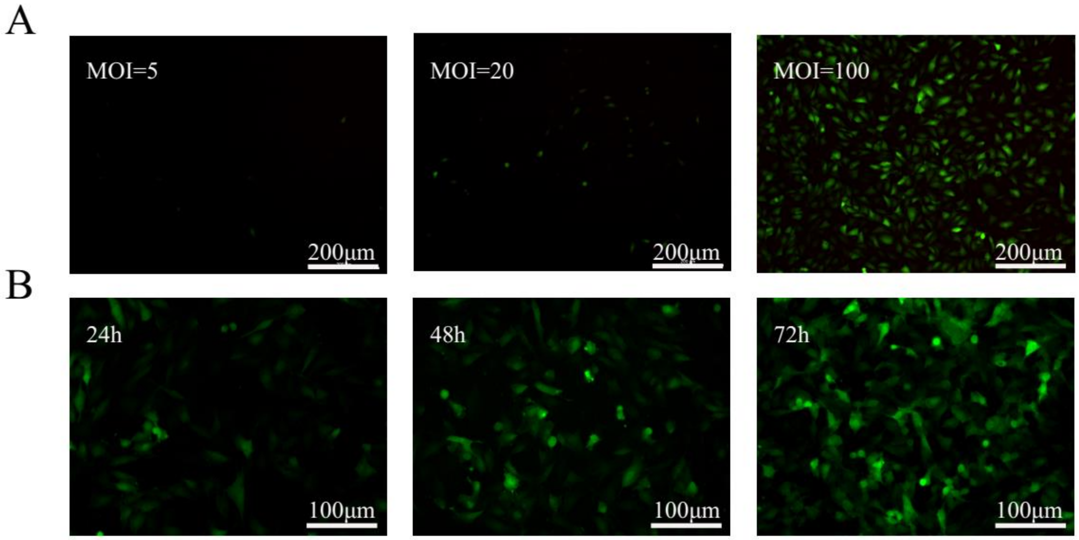

Supplementary Figure 2. In vitro determination of lentivector titre and transfection time. Cultured endothelial cells are seeded at 5 × 10^5 on 10 cm^2 plate and conducted viral vector transfection at 70 - 80% confluence. **(A)** endothelial cells transduced with different titre (MOI 5, 20, 100 respectively). Twenty-four hours after transduction, EGFP positive fluorescent cells were observed using fluorescent microscope (Olympus FV500, Japan) to evaluate proper MOI for cell transduction. **(B)** Assessment transduction efficiency of different lentivector incubation time (24, 48, 72 hours respectively) by density of fluorescent.

© 2018. Li T et al. Published online at http://www.aginganddisease.org/EN/10.14336/AD.2018.1001

SUPPLEMENTARY DATA

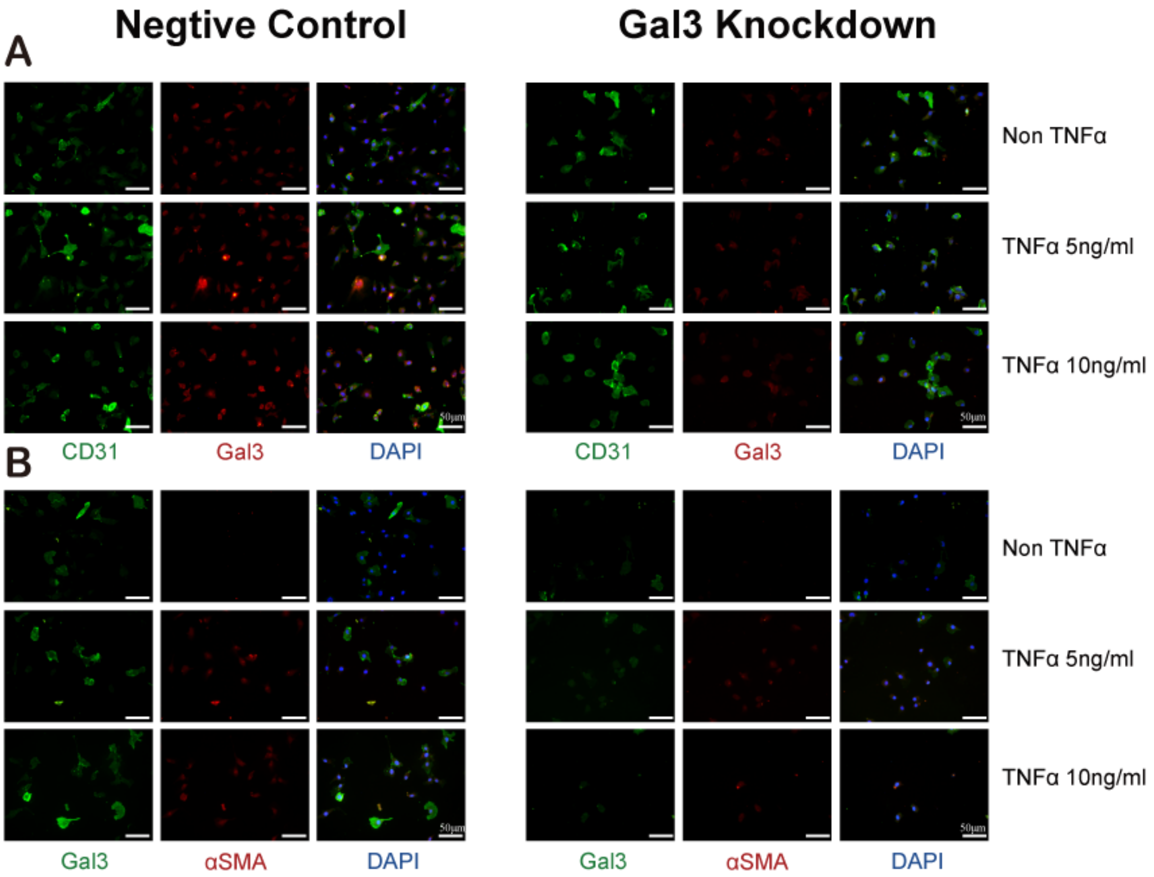

Supplementary Figure 3. Gal-3 siRNA partially reverse TNFα and Hypoxia leading to mesenchymal marker upregulation. Endothelial cells hardly expressed αSMA, while TNFα and Hypoxia enhanced αSMA expression. Gal-3 SiRNA (right) decreased injury stimulus caused αSMA upregulation.

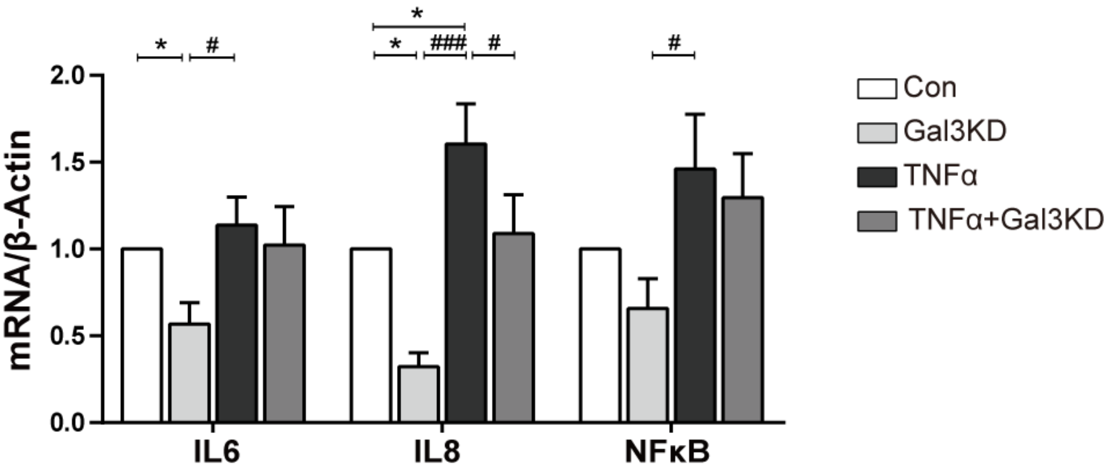

Supplementary Figure 4. Gal-3 siRNA partially inhibit TNFα induced inflammation cytokine. Gal-3 SiRNA reduced IL-8 upregulation induced by TNFα, IL-6 and NFκB have slight downregulation (not significantly). n=3, * indicate $p < 0.05$ comparing Control group; #, and ### indicate $p < 0.05$ and $p < 0.001$ respectively, comparing the TNFα administrated group Student's t-test was used for comparing differences in normoxia and hypoxia groups. Analyses performed by one-way ANOVA and Bonferroni post hoc.

© 2018. Li T et al. Published online at http://www.aginganddisease.org/EN/10.14336/AD.2018.1001
